# Antennal Development in the Praying Mantis (*Tenodera aridifolia*) Highlights Multitudinous Processes in Hemimetabolous Insect Species

**DOI:** 10.1371/journal.pone.0098324

**Published:** 2014-06-04

**Authors:** Thomas Carle, Yoshifumi Yamawaki, Hidehiro Watanabe, Fumio Yokohari

**Affiliations:** 1 Division of Biology, Department of Earth System Science, Faculty of Science, Fukuoka University, Fukuoka, Japan; 2 Department of Biology, Faculty of Science, Kyushu University, Fukuoka, Japan; University of Arizona, United States of America

## Abstract

Insects possess antennae equipped with a large number of segments (flagellomeres) on which sensory organs (sensilla) are located. Hemimetabolous insects grow by molting until they reach adulthood. In these species, the sensory structures develop and mature during each stage of development; new flagellomeres are generated at each molt elongating the antennae, and new sensilla appear. The praying mantis (*Tenodera aridifolia*) is a hemimetabolous insect with 7 different instars before it reaches adulthood. Because their antennae are provided with an atypical sensillar distribution, we previously suggested that their antennae develop with a different mechanism to other hemimetaboulous insect species. In the present study, we measured the number, length and width of flagellomeres along the antennae in nymph and adult mantis *Tenodera aridifolia*. For this study, we developed a new and innovative methodology to reconstruct the antennal development based on the length of flagellomeres. We observed and confirmed that the antennae of mantises develop with the addition of new segments at two distinct sites. In addition, we constructed a complete database of the features of the *flagellum* for each stage of development. From our data, we found that sexual dimorphism appears from the 6 instar (larger number and wider flagellomeres in males) in accordance with the appearance of their genital apparatus. The antennal sexual dimorphism completes at adulthood with longer flagellomeres and the emergence of a huge number of grooved peg sensilla in males during the last molting, which suggests once again their function as sex-pheromone receptive sensilla.

## Introduction

Animals evolved sensory structures that are adapted to their lifestyle. In insects, olfaction plays a pivotal role in detecting food and/or seeking sexual partners [1]. Many studies focus on species such as ants, moths, bees and cockroaches to understand the olfactory system because olfaction is crucial in their interactions. The praying mantis is a predator that relies more on their visual system [2]–[4]. Because of this reason, researchers focused on their vision [3], [4] as it shows the characteristics of binocular vision in mammals, and left the investigation of olfactory systems to be investigated in other insect species. However, the praying mantis, as with other insect species, also uses olfaction for feeding and courtship behaviors [5]–[7]. Moreover, their antennae present atypical features that might help to better understand the olfactory system [8].

Structurally, an antenna consists of a *scapus* (base), a *pedicellus* (stem) and a *flagellum*, which is divided into segments (flagellomeres) [1], and sensory organs (sensilla) that are located on their surface. Among other functions, some of these sensilla mediate olfactory information coming from odors or pheromones [1]. Mantises (*Tenodera aridifolia*) possess six different types of sensilla on their *flagellum*: chaetic (mechanoreception and contact chemoreception), campaniform (mechanoreception), coelocapitular (hygro- and thermoreception), basiconic, trichoid and grooved peg sensilla (olfaction) [8]. The sensillar distribution is not uniform along the *flagellum* in adults and varies within and between the sexes [8]. Because of this atypical sensillar distribution, we previously supported the idea of a specific and atypical form of antennal development in mantises compared to other hemimetabolous insects [8].

Hemimetabolous and holometabolous insects are distinguished due to differences in post-embryonic development. In hemimetabolous insects, there is no pupal stage and nymphs are similar to small adults without wings [9]. During the nymphal development, antennae elongates with addition of new flagellomeres. It has been generally assumed that this addition occurs at the proximal end of the *flagellum* at each molt [9]. For example, in grasshoppers and cockroaches, new flagellomeres appear at the proximal end by the division of the meriston (1 flagellomere) and by a binary division of a variable number of adjacent segments to the meriston, which are called meristal segments [10], [11]. In mantises (*Tenodera aridifolia*), the flagellar segments are not uniformly elongated along the antennae which suggests the presence of a second site where the flagellomeres divide during post-embryonic development e.g. [8]. This would constitute a different mechanism of antennal elongation, which might help to understand the general mechanism of antennal development in hemimetabolous insect species.

For this purpose, we investigated the antennal development in *Tenodera aridifolia* in the present study by measuring the features of the *flagellum* in nymphs and adults. We confirmed the presence of a second site of flagellar divisions active during post-embryonic development. In addition, we assembled a complete database of mantis antennae during development and revealed that sexual dimorphism appears at an earlier stage than that supposed in other insect species.

## Methods

### Insects and Breeding

Our observations were performed on mantises (*Tenodera aridifolia*). Oothecae were collected in a suburb of Fukuoka (Japan), grassland near Tachibana mountain (+33° 40′ 46.70″, +130° 28′ 6.20″). *Tenodera aridifolia* is neither an endangered nor protected species in Japan. No specific permission was required for collecting the oothecae at that site. Nymphs obtained from 4 oothecae were bred to adulthood using methods previously described [12], [13]. The mantises passed 7 different nymphean instars before reaching adulthood. The nymphs were painted dorsally in order to determine their molting as they were kept together until the 4/5 instars. After losing the paint on the carapace cuticle by molting, they were removed from the others, which had retained their painted markings, and were painted again. From the 6 instar, males and females were easily distinguished by their genitalia at the terminal abdomen, as in other insect species [14].

### Optical Microscopy

Antennae of adults were first observed using an optical microscope (BX50, Olympus) with an objective lens of X10. Micrographs of flagellomeres along antennae were taken along antennae and entire antennae were reconstructed by photomontage using GIMP (GNU software).

### Scanning Electron Microscopy

Antennae of adult mantises, and each instar of nymphs, were cut at the proximal part of the *scapus* and kept in a solution of acetone (50%). The isolated antennae were ultrasonically cleaned (Bransonic B1510, Yamato, Japan). As the nymphs did not have robust cuticular structures at early instars, the duration of washing varied depending on the instar but did not exceed 3 minutes. After washing, the antennae were dehydrated in an ascending series of acetone solutions (from 50 to 100%), air dried and then coated with platinum-palladium by an ion sputter (E-1045; Hitachi, Japan). Observations of antennae and digital image acquisitions were carried out using a field emission scanning electron microscope (S-4800; Hitachi, Japan), and the images were analyzed with GIMP (GNU software). The number of samples used for both microscopy and measurements are outlined in the tables. All measured values are expressed as mean ± standard error of the mean (SEM). Unpaired t-tests were used to compare the data using PSPP (GNU software).

## Results

### Sensillar Distribution

**Figure 1:**
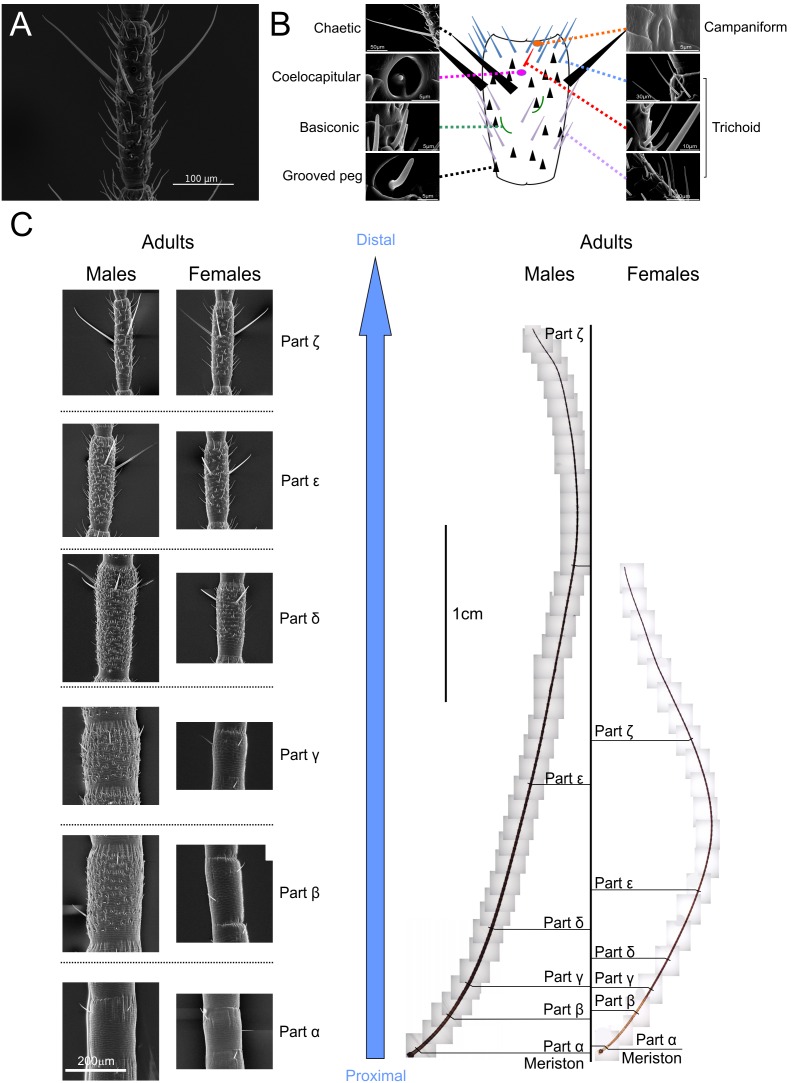
Sensillar distribution on flagellomeres (A and B) and along the antennae in adult mantises (*Tenodera aridifolia*) (C). SEM micrograph of a flagellomere (**A**) and its schematic representation (**B**). **C:** SEM Micrographs of flagellomeres in adults for both sexes along the antennae (left) and complete representation of antennae from optic micrographs (right). Based on an atypical sensillar distribution, the mantises’ antennae were divided into 6 distincts parts in adults (from α to ζ). The details of the sensillar identification and distribution are described briefly in results and completely in Carle *et al.* [8].

**Figure 2:**
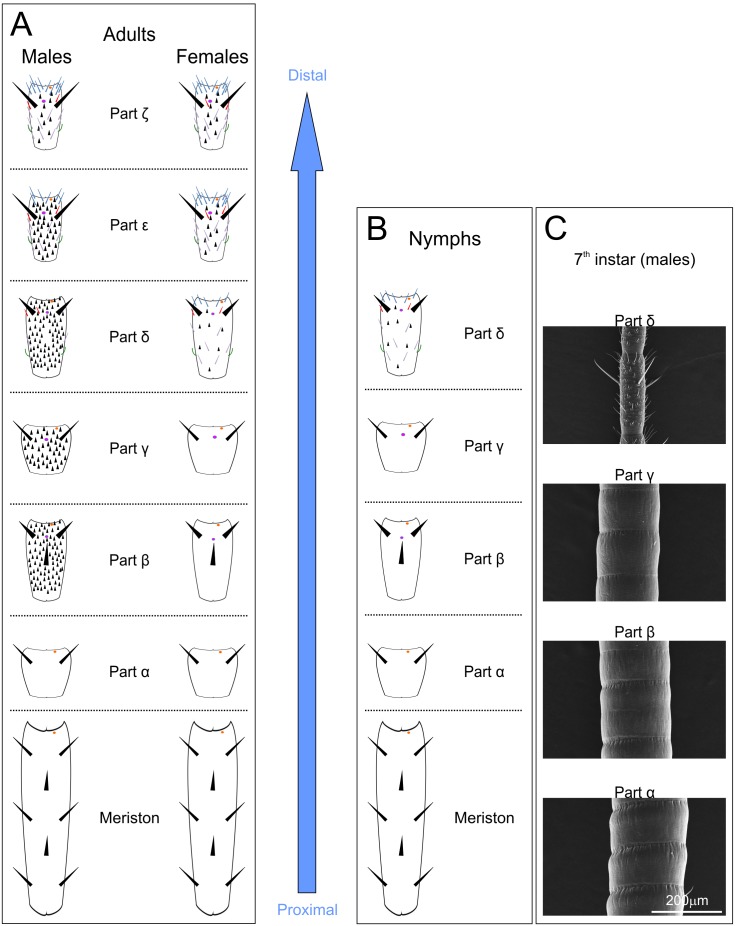
Sensillar distribution along the antennae in adults (A) and in nymphs (B and C) in *Tenodera aridifolia*. Depending on the sensillar distribution along the antennae, 6 different parts were distinguished in adults (from α to ζ) (**A**) but only 4 in nymphs (from α to δ) (**B**). **C:** Micrographs of the different parts in males at the 7^th^ instar.

As the sensillar distribution on flagellomeres (figures 1A–B) was not homogenous and varied along a longitudinal axis and between the sexes (figure 1C), we previously proposed a new division of the *flagellum* into 6 parts (α to ζ) in adults (figure 2A). The complete description was previously referenced e.g. [8], and is briefly summarized as follows:

#### Part α

Short flagellomeres with chaetic (in a single row), coeloconic and campaniform sensilla only.

#### Part β

Appearance of sexual dimorphism with a large number of grooved peg sensilla in male adults. In addition, chaetic sensilla are distributed into two circular lines in both sexes.

#### Part γ

A sudden reduction of the length of flagellomeres accompanied by a reduction in the number of circular lines of chaetic sensilla (from a double to a single line) is the criterion used to separate the parts γ φροµ β. All types of sensilla are distributed in an identical pattern as in the previous part.

#### Part δ

Trichoid and basiconic sensilla begin to occur in both sexes from the part δ. Moreover, grooved peg sensilla first appeared from this point in females. Nevertheless, whilst a large number of grooved peg sensilla dominated on males’ flagellomeres, trichoid sensilla are more numerous on females.

#### Part ε

The length of chaetic and trichoid sensilla suddenly increases from the part ε in both sexes. In the part ε, all types of sensilla are distributed in an identical pattern as the previous part.

#### Part ζ

Although grooved peg sensilla are dominant on the flagellomeres from the parts β to ε in males, both sexes presented a similar pattern of sensillar distribution in the part ζ.

The sensillar distribution of nymphs differs from that of adults due to the absence of a large number of grooved peg sensilla that are present in male adults (figures 2B–C). Thus, we reduced the nymphal flagellar partition by taking into account only the parts α to δ with the same criteria as previously used in adults [8]. In this nymphal partition, the part δ is more generally defined, similarly for adult females, by the appearance of olfactory sensilla that are not present on the more proximal parts.

### Widths

**Figure 3:**
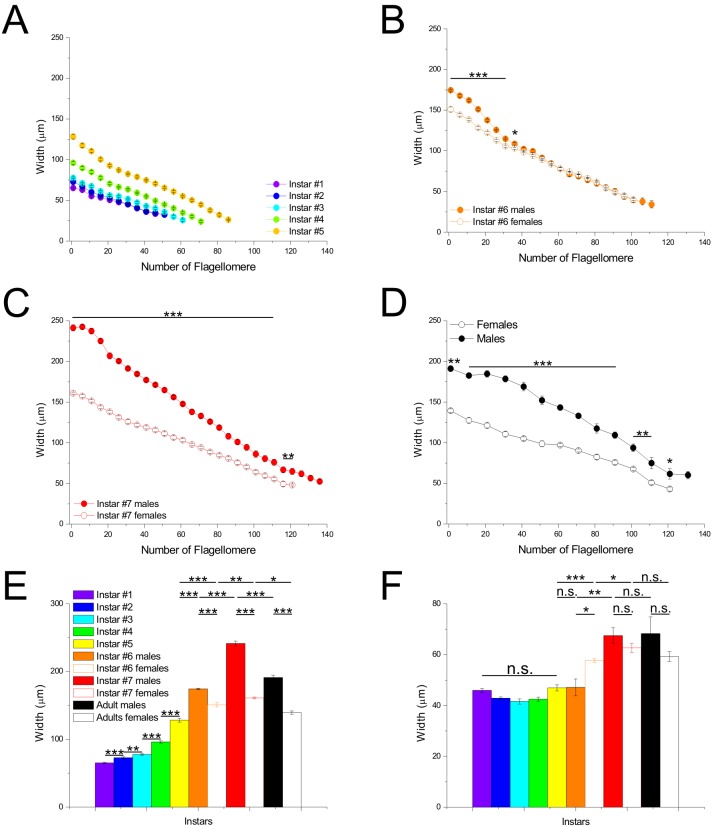
Widths of antennae depending on flagellomere location. Curves of width in nymphs from the 1^st^ to the 5^th^ (**A**), at the 6^th^ (**B**) and 7^th^ (**C**) instars and in adults (**D**). In **B to D**, males are represented with filled circles and females with empty circles. **E:** Width of the 1^st^ flagellomere for the different instars and genders. **F:** Width at the distal part of antennae (average of flagellomeres located between the 10^th^ and 30^th^ flagellomeres from the distal end) for the different instars and genders. Differences are marked as: n.s.: p>0.05; *: p<0.05; **: p<0.01; ***: p<0.001.

The width of flagellomeres, which reflected the diameter of antennae, gradually decreased from the proximal to the distal part of *flagellum* in a uniform manner (figure 3). Until the 6^th^ instar, we did not observe any noticeable differences in width between the respective flagellomeres between the sexes (figures 3A–B). In contrast, in the 7^th^ instar and in adults, males clearly possessed wider flagellomeres than females along the antennae except at the distal end (figures 3C–D).

The 1^st^ flagellomere (at the base of the *flagellum*) increased in width in concurrence with molts (figure 3E). However, even if differences appeared between the 1^st^ and 2^nd^ instars (t_22.5_ = −5.148, p<0.001), these were small until the 3^rd^ instar (about 13µm between the 1^st^ and 3^rd^ instar) (table 1). The 1^st^ flagellomere was greatly enlarged by the 4^th^ instar (about 18µm between the 3^rd^ and 4^th^ instars), though the number of flagellomeres added (about 11) was fewer than that at the transition between the 1^st^ and 3^rd^ instars (about 14). The appearance of abdominal sexual dimorphism at the 6^th^ instar was accompanied by an enlargement of the 1^st^ flagellomere in males compared to females (figure 3E; t_9_ = −6.727, p<0.001) and more notably at the 7^th^ instar (t_18_ = −18.963, p<0.001). Such sexually dimorphic enlargement of the 1^st^ flagellomere was not observed in younger nymphs before the 6^th^ instar by looking at the individual curves. Surprisingly, the width of the 1^st^ flagellomere was reduced during the last molting. The reduction was more noticeable in males (Δ = 50.2 µm; t_16_ = 9.284, p<0.001) than in females (Δ = 21.6 µm; t_13_ = 9.106, p<0.0001).

**Table 1:** Widths of flagellomeres during post-embryonic development.

				Width (µm)																											
				Number of flagellomere																											
	Instars			#1	#6	#11	#16	#21	#26	#31	#36	#41	#46	#51	#56	#61	#66	#71	#76	#81	#86	#91	#96	#101	#106	#111	#116	#121	#126	#131	#136
	#1		**Mean**	**65,2**	**63,0**	**55,3**	**53,5**	**50,5**	**47,8**	**45,3**	**40,3**	**36,1**	**33,5**																		
	(n = 11)		SEM	0,8	1,1	1,0	1,0	1,1	0,9	1,2	0,6	0,7	0,6																		
	#2		**Mean**	**72,8**	**67,0**	**60,2**	**56,1**	**53,4**	**49,0**	**45,2**	**40,8**	**36,3**	**34,5**	**32,5**																	
	(n = 14)		SEM	1,2	1,2	1,0	1,0	1,0	0,6	0,7	0,5	0,6	0,5	0,7																	
			Stats #1-#2	***							**																				
	#3		**Mean**	**77,7**	**71,0**	**67,3**	**61,1**	**56,1**	**54,8**	**51,6**	**47,2**	**42,9**	**40,1**	**35,6**	**30,0**	**25,6**															
	(n = 16)		SEM	1,0	1,0	1,1	0,9	0,9	0,9	1,0	1,1	0,9	1,2	1,1	1,6	1,4															
			Stats #2-#3	**									-																		
	#4		**Mean**	**96,0**	**89,7**	**84,9**	**77,7**	**70,5**	**66,4**	**63,5**	**59,2**	**54,9**	**49,5**	**44,9**	**40,3**	**34,8**	**30,2**	**23,8**													
	(n = 17)		SEM	1,8	1,6	1,7	1,6	1,3	0,8	0,8	0,7	0,8	0,8	0,7	0,9	1,2	1,3	1,3													
			Stats #3-#4	***											-																
	#5		**Mean**	**128,4**	**117,5**	**110,5**	**100,3**	**92,7**	**87,1**	**82,5**	**78,9**	**74,8**	**70,6**	**65,4**	**60,8**	**55,2**	**50,2**	**44,8**	**37,7**	**32,1**	**26,2**										
	(n = 17)		SEM	2,9	2,1	1,8	1,7	1,3	1,0	0,9	1,1	0,8	1,0	1,1	1,0	1,1	1,0	1,3	1,5	1,4	0,6										
			Stats #4-#5	***														**													
	#6		**Mean**	**174,5**	**167,8**	**162,0**	**151,1**	**137,7**	**125,7**	**114,7**	**108,6**	**102,2**	**99,5**	**91,3**	**84,6**	**77,8**	**71,4**	**68,7**	**64,2**	**60,0**	**54,9**	**50,1**	**43,9**	**39,6**	**37,8**	**34,1**					
	(n = 5)		SEM	1,1	1,6	1,1	1,4	1,4	0,9	1,1	1,5	2,0	1,9	2,2	2,6	2,1	2,9	2,9	2,8	3,2	3,2	3,6	3,4	3,3	4,2	4,5					
			Stats #5-#6	***																			-								
	#7		**Mean**	**241,4**	**242,6**	**237,3**	**225,1**	**206,9**	**200,4**	**191,3**	**184,7**	**177,1**	**171,3**	**164,9**	**156,2**	**147,7**	**138,0**	**133,0**	**126,0**	**118,7**	**108,0**	**101,0**	**94,4**	**86,2**	**80,5**	**76,1**	**66,8**	**64,8**	**61,8**	**56,6**	**52,5**
Males	(n = 10)		SEM	3,7	2,9	3,4	2,6	3,7	1,9	1,7	2,1	2,1	1,6	2,5	2,0	2,2	3,2	2,6	2,5	2,8	3,6	3,1	3,6	4,0	3,8	3,7	3,5	3,8	3,7	3,5	2,8
			Stats #6-#7	***																								**			
	Adults		**Mean**	**191,2**		**182,7**		**184,7**		**178,5**		**169,1**		**152,2**		**143,3**		**133,0**		**117,5**		**109,3**		**93,8**		**74,9**		**61,7**		**60,2**	
	(n = 7)		SEM	3,5		3,0		4,0		3,7		5,3		5,4		3,1		2,0		5,6		3,9		4,7		6,9		6,4		4,8	
			Stats #7-#A	***																								-			
	#6		**Mean**	**150,9**	**144,2**	**138,5**	**128,2**	**122,3**	**112,9**	**105,8**	**103,2**	**98,4**	**94,2**	**89,2**	**83,7**	**77,9**	**74,4**	**70,9**	**66,3**	**61,7**	**54,1**	**48,8**	**44,9**	**40,2**							
	(N = 6)		SEM	3,0	1,5	1,3	1,4	1,7	1,3	1,3	1,2	0,8	0,9	1,1	0,7	1,5	0,6	0,3	0,3	0,8	0,9	0,9	1,2	1,1							
			Stats #5-#6	***																	***										
			Stats M-F	***																	*										
	#7		**Mean**	**161,0**	**157,4**	**151,5**	**143,6**	**138,2**	**131,3**	**125,9**	**122,0**	**119,0**	**115,7**	**111,4**	**106,5**	**103,2**	**98,0**	**94,1**	**88,6**	**84,7**	**80,9**	**75,4**	**70,3**	**64,0**	**59,6**	**55,6**	**49,4**	**48,1**			
Females	(n = 9)		SEM	1,2	1,8	1,4	1,7	1,6	1,8	2,4	1,9	1,9	2,2	2,2	2,0	1,9	1,5	1,6	1,7	1,8	1,8	2,2	1,9	2,0	2,1	1,8	2,9	1,2			
			Stats #6-#7	**																					*						
			Stats M-F	***																					-						
	Adults		**Mean**	**139,4**		**127,5**		**121,2**		**110,6**		**105,0**		**98,7**		**97,1**		**90,4**		**82,4**		**75,8**		**67,7**		**50,8**		**43,1**			
	(n = 7)		SEM	2,3		3,2		3,0		3,1		2,2		3,5		1,7		1,8		2,7		2,5		1,7		2,5		2,5			
			Stats #7-#A	*																					-						
			Stats M-F	***																					-						

The boxes represent the means that were averaged in order to compare the widths of antennae at the tip end (between the 10 and 30 flagellomeres) between the different instars (see Results section). Differences between the boxes are marked as: -: p > 0.05;

*: p < 0.05;

**: p < 0.01;

***: p < 0.001.

We examined the changes in width of flagellomeres at the distal parts of antennae from the 1^st^ instar to adulthood by averaging the width between the 10^th^ and 30^th^ flagellomeres by starting to count from the antennal tip (figure 3F). We found that the width was constant from the 1^st^ to the 5^th^ instar (instar #1: 46±0.8 µm, instar #5: 47±1.2 µm, t_9_ = 0.603, p>0.05). Nevertheless, females showed an enlargement of the distal parts of antennae during the transition from the 5^th^ to the 6^th^ instars (instar #5: 47±1.2 µm, instar #6: 57.7±0.7 µm, t_9_ = −5.154, p<0.001), and such an enlargement occurred in males between the 6^th^ to the 7^th^ instars only (instar #6: 47.1±3.3 µm, instar #7: 67.5±3.1 µm, t_9_ = −4.084, p<0.001). Nevertheless, the width was neither different between males and females at the 7^th^ instar (males: 67.5±3.1 µm, females: 62.7±5.2 µm, t_9_ = 1.324, p>0.05) nor at adulthood (for all values; t<1.322, p>0.05).

### Lengths

**Figure 4:**
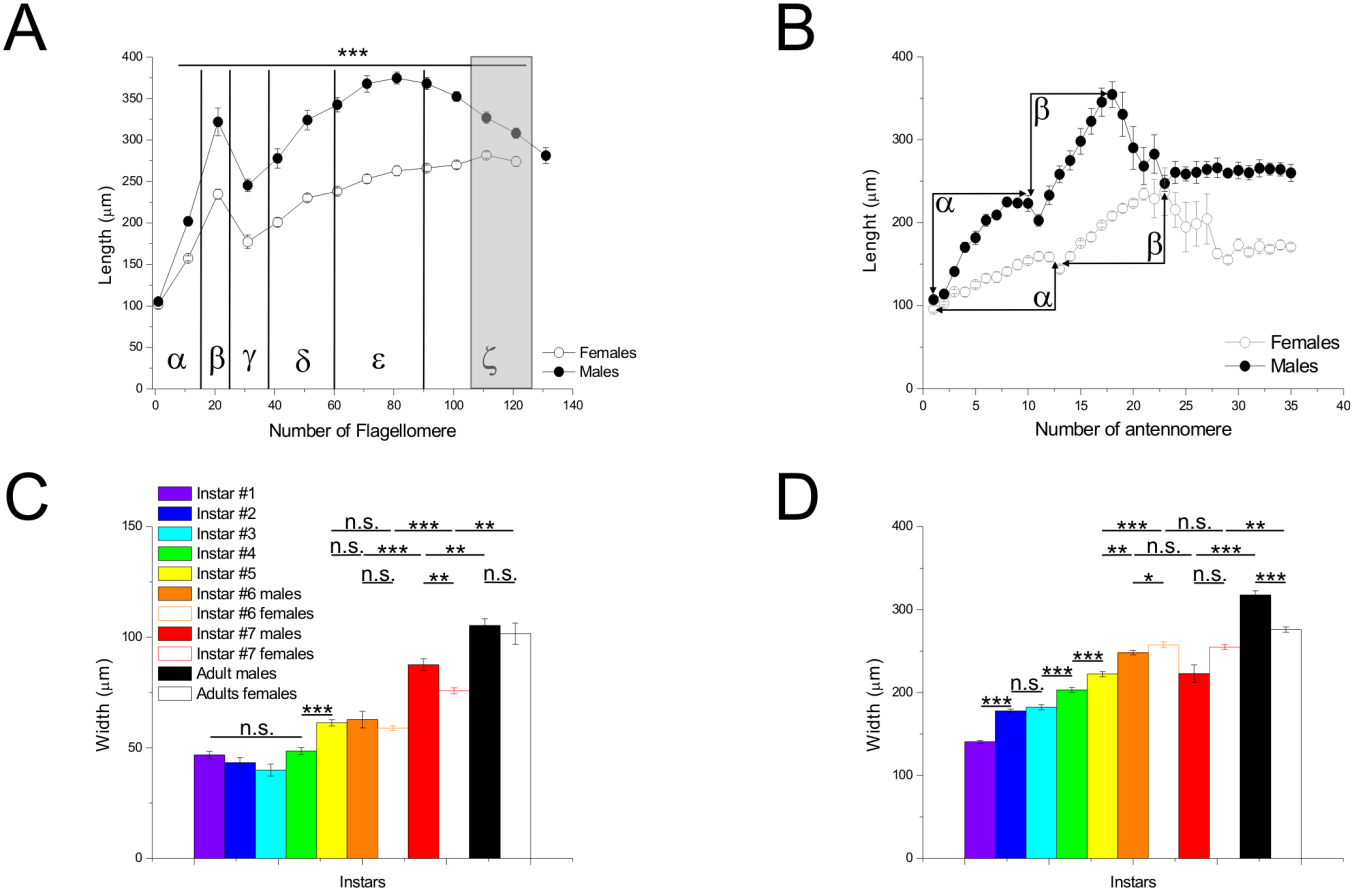
Lengths of flagellomeres. A–B: The graphs represent the lengths of flagellomeres measured every 10 (**A**) or every (**B**) segment from the proximal part of antennae in male (filled circles) and female (empty circles) adults. **C–D:** Length of the 1^st^ flagellomere and at the distal part of antennae for the different instars and genders as in figure 3.

In adults, the antennae showed obvious differences in length between the sexes (figure 1C; males: 39.9±0.1 mm, females: 29.8±0.4 mm, t_12_ = −27.437, p<0.0001). In order to elucidate the morphological features of the mantises’ antennae, we examined the number and length of the flagellomeres of both sexes. From our measurements, we observed a significant difference in number between the sexes (t_7.147_ = −3.074, p = 0.017) (table 2). In addition, every corresponding flagellomere was significantly longer in males than females (for all values t<−3.634, p<0.01), with the exception of the most proximal segment (t_12_ = −0.656, p = 0.524). The curves of length of flagellomeres were not uniform but the patterns of change were fairly similar between the sexes (figures 4A–B): a gradual increase from flagellomeres #1 to #10/15 reaching an asymptote, a second increase until a peak around flagellomere #20/25, and then a sudden decrease (valley) in both sexes. After the sudden reduction, the length gradually increased with uniformity until the antennal tip segment in females, while it increased to a larger degree in males before a gradual diminution from about flagellomere #70. The pattern of change of the length of flagellomeres was similar in nymphs (figures S1-S2).

**Table 2:** Lengths and number of flagellomeres during post-embryonic development.

			Length (µm)																												Total numberofFlagellomeres
			Number of flagellomere																												
	Instars		#1	#6	#11	#16	#21	#26	#31	#36	#41	#46	#51	#56	#61	#66	#71	#76	#81	#86	#91	#96	#101	#106	#111	#116	#121	#126	#131	#136	
	#1	**Mean**	**46,7**	**72,9**	**114,0**	**91,4**	**126,0**	**143,5**	**144,3**	**147,8**	**138,2**	**128,6**																			48,3
	(n = 11)	SEM	1,7	2,0	7,9	2,6	3,0	1,2	2,3	2,1	6,0	5,4																			1,7
	#2	**Mean**	**43,2**	**68,7**	**114,4**	**176,1**	**124,0**	**152,2**	**176,6**	**188,6**	**193,7**	**185,7**	**180,9**																		54,0
	(n = 14)	SEM	2,3	1,8	2,5	4,5	2,4	2,8	2,2	2,0	1,4	1,9	2,8																		0,6
		Stats #1-#2	-							***																					**
	#3	**Mean**	**39,9**	**48,1**	**72,2**	**112,3**	**110,4**	**104,2**	**129,0**	**155,3**	**171,9**	**193,9**	**209,2**	**223,2**	**220,5**																62,1
	(n = 16)	SEM	2,7	1,1	3,5	3,0	8,6	2,8	3,7	2,8	2,8	4,3	4,1	5,3	4,1																0,8
		Stats #2-#3	-									-																			***
	#4	**Mean**	**48,5**	**56,4**	**75,7**	**116,5**	**145,5**	**105,3**	**123,8**	**142,4**	**160,9**	**180,0**	**197,1**	**209,2**	**227,1**	**234,9**	**258,2**														73,8
	(n = 17)	SEM	1,6	1,3	2,9	3,6	3,2	3,4	3,0	2,8	3,3	3,4	3,6	5,5	3,6	5,5	2,2														1,2
		Stats #3-#4	**											***																	***
	#5	**Mean**	**61,3**	**66,7**	**76,0**	**115,7**	**136,9**	**138,4**	**107,7**	**125,0**	**138,1**	**150,7**	**164,8**	**180,9**	**196,1**	**215,1**	**227,8**	**250,0**	**252,1**	**274,3**											90,2
	(n = 17)	SEM	1,4	1,7	3,1	2,4	2,1	6,9	4,1	4,2	3,8	4,6	4,4	4,2	4,3	3,9	4,1	4,5	4,0	3,3											1,3
		Stats #4-#5	***														***														***
	#6	**Mean**	**62,8**	**73,9**	**85,0**	**102,7**	**127,4**	**145,2**	**165,5**	**159,6**	**118,0**	**124,0**	**137,5**	**143,7**	**164,8**	**187,1**	**201,8**	**213,8**	**224,1**	**236,4**	**243,7**	**256,0**	**256,2**	**266,1**	**273,2**						**111,2**
	(n = 5)	SEM	3,7	6,0	3,1	7,8	4,2	6,8	6,7	20,0	4,0	4,5	4,5	5,7	5,0	1,7	2,2	4,4	5,1	3,5	5,6	2,3	3,9	1,7	3,0						1,7
		Stats #5-#6	-																			***									***
	#7	**Mean**	**87,6**	**116,1**	**140,1**	**168,0**	**172,8**	**117,7**	**125,6**	**131,0**	**135,8**	**141,1**	**158,5**	**166,2**	**170,3**	**176,0**	**184,6**	**190,9**	**194,8**	**210,0**	**210,8**	**219,1**	**221,6**	**230,2**	**237,3**	**248,7**	**243,7**	**246,3**	**258,7**	**264,2**	**137,2**
Males	(n = 10)	SEM	2,7	2,9	4,5	4,8	18,5	4,6	5,9	4,6	5,5	5,4	5,8	6,3	5,4	6,7	3,9	4,4	4,9	6,3	4,3	5,9	3,8	4,9	5,1	6,8	6,0	5,8	5,7	4,1	3,5
		Stats #6-#7	***																								-				***
	Adults	**Mean**	**105,3**		**202,0**		**321,9**		**245,4**		**277,8**		**324,1**		**342,5**		**367,8**		**374,7**		**368,2**		**352,5**		**326,8**		**308,2**		**281,3**		**130,7**
	(n = 7)	SEM	3,1		5,4		16,6		7,4		11,8		11,7		8,7		10,0		7,2		7,4		5,7		6,9		6,2		9,4		2,9
		Stats #7-#A	**																								***				-
	#6	**Mean**	**58,9**	**66,0**	**77,9**	**94,5**	**122,6**	**146,1**	**168,3**	**229,9**	**128,3**	**140,8**	**152,7**	**166,3**	**182,9**	**213,2**	**227,9**	**239,9**	**252,5**	**261,9**	**275,4**	**282,3**	**282,4**								**103,5**
	(n = 6)	SEM	1,1	2,5	1,1	4,2	2,1	2,2	2,2	3,5	2,1	1,8	3,5	2,9	4,8	4,4	3,4	2,9	1,7	7,4	3,0	5,1	6,2								1,1
		Stats #5-#6	-																	***											***
		Stats M-F	-																	*											**
	#7	**Mean**	**75,8**	**90,6**	**110,9**	**140,4**	**183,8**	**210,0**	**118,5**	**134,1**	**140,4**	**157,6**	**166,5**	**173,0**	**181,8**	**192,9**	**203,9**	**216,0**	**222,8**	**234,2**	**237,2**	**244,8**	**252,5**	**261,3**	**261,7**	**275,2**	**264,3**				**125,2**
Females	(n = 9)	SEM	1,4	3,8	2,8	5,4	5,2	7,8	4,1	4,9	5,9	5,3	4,5	5,4	6,2	5,2	5,3	5,1	5,4	3,7	5,8	5,1	5,3	5,9	6,5	6,8	6,1				4,5
		Stats #6-#7	***																					-							**
		Stats M-F	**																					-							*
	Adults	**Mean**	**101,6**		**157,3**		**234,8**		**177,4**		**200,9**		**230,2**		**238,1**		**253,2**		**262,8**		**266,2**		**270,0**		**281,8**		**274,1**				**121,4**
	(n = 7)	SEM	4,8		3,0		5,9		8,1		5,5		4,3		4,1		5,3		6,1		3,7		4,6		3,9		5,9				0,9
		Stats #7-#A	***																					**							-
		Stats M-F	-																					***							*

An explanation for the boxes is reported in the legend of table 1.

-: p > 0.05;

*: p < 0.05;

**: p < 0.01;

***: p < 0.001.

At the base of antennae (figure 4C), we observed an elongation of the 1^st^ flagellomere between the 4^th^ and 5^th^ instars (t_32_ = −6.065, p<0.001) and after the 6^th^ instar (for all values; t <−6.508, p<0.001). Although males and females presented similar lengths for flagellomeres #1 at the 6^th^ instar and in adults (for all values; t <−0.656, p>0.05), there was a significant difference difference at the 7^th^ instar between the sexes (t_11.262_ = −4.697, p<0.01).

At the distal part of antennae (figure 4D), the length of flagellomeres gradually increases during each molt (for all values; t <−3.045, p<0.01), except between the 2^nd^ and 3^rd^ instars, and between the 6^th^ and 7^th^ instars (for all values; t<1.337, p>0.05). Although a significant difference occurs between the sexes at the 6^th^ instar (males: 248.1±2.7 µm, females: 257.4±2.7 µm, t_9_ = −2.426, p<0.01), the flagellomeres undergo a marked sexual dimorphism by the time they reach adulthood, with longer flagellomeres in males (males: 317.5±6.3 µm, females: 275.9±3.9 µm, t_12_ = 5.587, p<0.001).

### Flagellomere Addition

**Figure 5:**
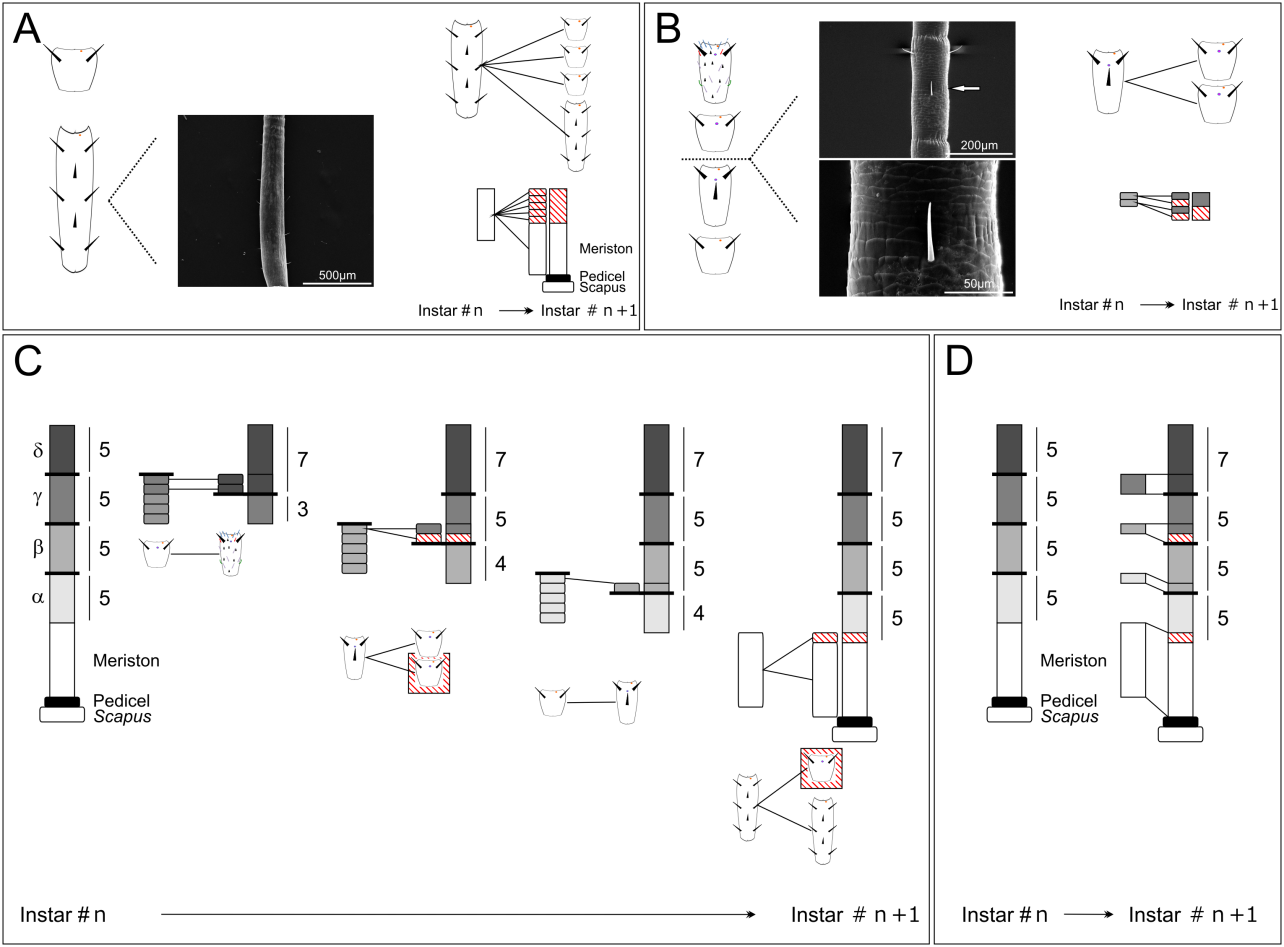
Schematic representation of the genesis of new flagellomeres and expansion of antennae in mantis. **A:** A part of flagellomeres newly formed during molting is generated at the proximal end of antennae by the division of the meriston, which presents many rows of chaetic sensilla as shown on the SEM micrographs. B: Looking at the features of flagellomeres along the antennae, another site of genesis lies at the transition between the parts β and γ, where a single flagellomere of the part β divides to give two flagellomeres of the part γ after molting. On the right side, the mechanism of genesis is illustrated by two schematic representations. **C and D: Mechanism of expansion of antennae.** This model, starting with 5 segments in each part, details the process of antennal development with an expansion of the part δ of 2 flagellomeres. The different transitions are represented (**C**) and the number of flagellomeres composing each part is indicated on the right of each representation. Red lines on white background represent an increase in the total number of flagellomeres. **D:** Similar representation as **C** without details.

At the proximal parts of antennae in nymphs and adults, the first flagellomere (meriston) was long and always equipped with several rows of chaetic sensilla, while the neighboring segments were very short and increased distally in length (figures 4A–B, 5A). These features have been observed in other hemimetabolous insects, such as cockroaches [15]. It is known in cockroaches that the meriston divides into multiple flagellomeres (meristal segments) at a given molt and these meristal segments divide into two flagellomeres at the next molt. However, in mantises new flagellomeres appear to be formed not only in a similar fashion but also in an additional fashion as stated below. As observed in every instar and in adults, the part β was composed of long flagellomeres possessing two rows of chaetic sensilla, while the part γ was composed of short flagellomeres equipped with a single row of chaetic sensilla [8]. Additionally, in both sexes we observed partial divisions in a transitional flagellomere between these two parts (figure 5B). These suggested that the flagellomeres of the part γ were born from a simple binary division of the flagellomeres coming from the part β during postembryonic development.

Based on this mechanism, we created a schematic example of antennal development. The figures 5C–D show an increase in the number of flagellomeres in the part δ from 5 to 7, with the other parts (α-γ) maintaining a composition of 5 flagellomeres. In this example, the antenna would develop as follows: an increase of 2 flagellomeres of the part δ would mean that 2 flagellomeres from the part γ developed olfactory sensilla and become flagellomeres of the part δ (expanding this last part). This change would directly induce modifications on the more proximal parts. Thus, to keep the part γ constant, a single flagellomere from the part β would divide to give two flagellomeres henceforth belonging to the part γ and which would compensate for the two flagellomeres that matured. Thus, a single flagellomere maturing from the part α (by increasing in size and developing a second row of chaetic sensilla) would maintain the number of flagellomeres of the part β. Finally, to keep part α constant, the meriston would produce a single flagellomere in addition to a new meriston.

### New Methodology for the Investigation of Antennal Development

Mapping antennal development in hemimetabolous insects possessing a large and inter-individually variable (between individuals) number of flagellomeres requires a specific methodology. The general mechanism of antennal development can be defined only by the observation of antennae at the different instars in species with a small number of antennal segments, such as grasshoppers and mantophasmids [11], [16]. However, since there are inter-individual differences in hemimetabolous insect species such as cockroaches or mantises that possess many flagellar segments and grow with many molts, basic observation is not sufficient to elucidate the general mechanism of antennal development of these insects [10]. In this case, it is required to average the number of new flagellomeres added because of inter-individual variations. In mantises (*Tenodera aridifolia*), antennae develop by the addition of flagellomeres at two distinct sites (figure 5). In this case, simple observations were not sufficient to delineate the transitions between the different parts. For example, it is difficult to observe a clear transition between parts α and β because the length of flagellomeres increases in a gradual manner and the appearance of sensilla is not homogenous. However, measuring the length of flagellomeres clearly marks the transition between these two parts (figure 4B). In this way, we solved an observational problem using a mathematical method that could be generalized to other hemimetabolous insects possessing a large number of flagellomeres.

**Figure 6:**
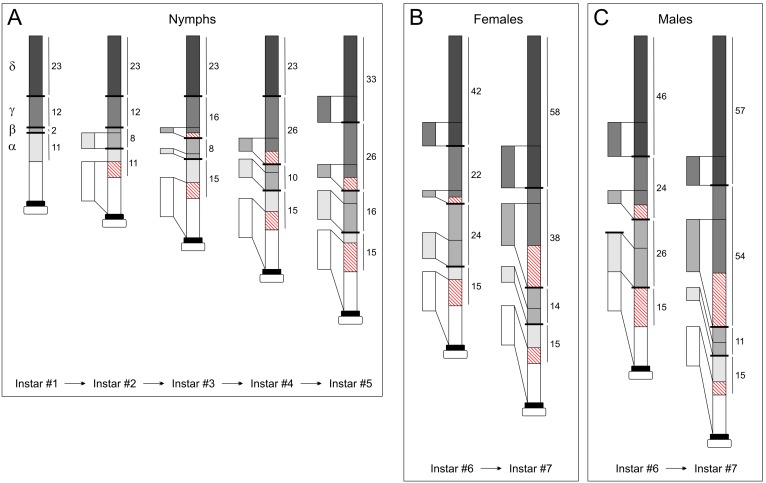
Antennal development in mantis in nymphs (A–C) following the scheme of **figure 5D** and **table 2**.

By taking advantage of the features of the curves of length (figure S3A), we investigated and generalized the antennal growth in mantises. During postembryonic development, the number of flagellomeres increased in an irregular manner (see table 2). Consequently, these features more or less shifted the curves obtained on the following instars after molting. This enabled us to conceive four models of antennal elongation (figure S3). First, a right movement of the valley represents an expansion of the parts α and/or β (model A) because the valley marks the transition between the parts β and γ (figure S3B). Since the length of flagellomeres in the part α reaches an asymptote (figure 4B), we can unambiguously identify the transition point between the parts α and β. Therefore, an addition of segments in the part α elongates the part of the curve lower to the horizontal dot line, and the slope of the curve obtained after molting is reduced as shown in model 1. Conversely, an increase of the number of flagellomeres of the part β expands the part of the curve upper to the horizontal dot line, involving no change of the slope of the curve as shown in model 2. Second, by normalizing the curves from the distal end of the antennae, a left shift of the valley represents an increase of the parts γ and/or δ (model B, figure S3C). In this case, two models (models 3 and 4) are applicable: a simple left shift of the valley corresponds to an expansion of the part γ (model 3) while a left shift of the black triangle corresponds to a growth of the part δ, and *a fortiori* to an increase in the number of flagellomeres possessing olfactory sensilla (model 4). We measured the lengths of flagellomeres every 5 segments (figures S1-S2), and based on these four models we analyzed the curves normalized from the proximal and distal parts of antennae to investigate the expansions and/or reductions of the different parts. With this method, we succeeded in describing postembryonic antennal development (figure 6). Detailed descriptions are available in the appendix SI.

## Discussion

By looking precisely at the morphological features of flagellomeres, such as their length and width, we were able to understand many aspects of antennal development in mantises. In particular, those features decisive for the flagellar partition that we had previously proposed allowed us to develop a specific methodology to investigate antennal development. Based on this, we highlighted that the antennae of mantises develop in a manner that is partially understood in other hemimetabolous insect species and might uncover new information about a new or a general mechanism of flagellar addition. Moreover, we found that antennal sexual dimorphism begins to appear in nymphs in two steps. The first step is related to a higher increase in width and a higher number of flagellomeres in males at the 6^th^ instar. The second step occurs during the last molting with an elongation of flagellomeres and the appearance of a huge number of grooved peg sensilla in males, which undoubtedly constitutes their sex-pheromone receptive sensilla. Incidentally, this type of sensilla has never been referenced as a sex-pheromone receptive sensilla in other insect species, and it is a rare case being a double-walled sensilla having this function e.g.[8].

### Antennal Development

Although cockroaches, mantophasmids, crickets and mantises are phylogenetically closely related species, the features of their antennal development differ considerably. The mantises have a second site, which is absent in cockroaches, where new flagellomeres are added and distinct from the site composed of the meriston and the meristal segments. The presence of this second site was previously schematized in grasshoppers during their early development (1^st^ to 3^rd^ instars) and referred to in mantophasmids in adulthood, though little attention was paid to their ontogenesis [11], [16]. The antennal developing pattern shown in our study suggests that there might be a new mechanism of antennal development that should be examined in future research.

The number of new flagellomeres formed from the meriston is irregular during postembryonic development in mantises and more generally in insects possessing a large number of flagellomeres. This number increases until the 6^th^ instar and then regresses, as observed in the cockroach species *Periplaneta americana* [17] and *Leucophaea maderae* [10]. The meriston divides into between 4 and 14 new segments at each molt in *Periplaneta americana*, similar to *Tenodera aridifolia* (between 5 and 15). In contrast, the meriston consistently generates a single new meristal segment at each molt in species with a fewer number of flagellomeres, such as grasshoppers and mantophasmids [11], [16]. In these phylogenetically close species, grasshoppers and mantophasmids possess 24 and 14 flagellomeres at adulthood, respectively, while mantises and cockroaches have more than one hundred. This difference might explain the higher production of new segments by the meriston in the latter two species but cannot explain the irregular number produced that we observed.

The flagellomeres also evolve in length and width during postembryonic development. Every flagellomere lengthens (figure 4D) at each molt as in locusts [11]. However, data on the diameter of antennae appear to be scarce in previous studies of antennal development. In the present study, although the width of the respective flagellomeres is constant at the distal part of the antennae until the 5^th^ instar, we observed an increase at the 6^th^ instar in females and at the 7^th^ instar in males. Unfortunately, the reason for the enlargement of distal segments or the sexual difference in the time course of antennal development is as yet unclear. The width at the flagellar base increases somewhat from the 1^st^ to 3^rd^ instars and obviously from the 4^th^ until the 7^th^ instar. However, we are unable to compare the features to those of other insects, as there have been few studies on developmental changes of antennal diameter in any insect species, although the diameter of the base tends to increase with the appearance of new flagellomeres according to Chapman [11].

### Appearance of Sexual Dimorphism

The antennal sexual dimorphism of antennae seems to be synchronized with the abdominal differentiation in *Tenodera aridifolia*. In some species of insects, including mantises, a good method of distinguishing between males and females before adulthood is to observe the abdomen [18]–[20], more specifically the abdominal segmentation [21]–[23]. In *Tenodera aridifolia*, this differentiation is visible from the 6^th^ instar. In parallel, we were unable to distinguish any sexual dimorphism of antennae (in length or width) until the 5^th^ instar from the individual’s curves (data not shown). Interestingly, the synchronized appearance of abdominal and antennal sexual dimorphism suggests induction through a similar factor. It is well known that ecdysone is directly implied in antennal development and especially in molting in insects [24], [25]. Moreover, its role is not only associated with development but has recently also been linked to sexual dimorphism. Examining the expression of receptors for ecdysone in antennal cells might answer this supposition and be an interesting point for future research.

During the last molting, sexual dimorphism is strongly marked in mantises with differences in term of lengths of flagellomeres and the sensillar distribution. Males develop larger flagellomeres provided with a huge number of grooved peg sensilla. In many insect species, males develop sex-pheromone-receptive sensilla during the last molting as in cockroaches [26], [27]. Functionally, even if sexual dimorphism appears at an early stage, males can only search for females at adulthood when the intersex genitalia are in place. In other words, there is no advantage to search (or to possess structures to search) for a sexual partner if the genital apparatus is not completely formed. So, the fact that a huge number of grooved peg sensilla appear during the last molting in male mantises undoubtedly confirms that this type of sensillum is involved in sex-pheromone-detection in mantises. This constitutes the unique case of double-walled sensilla which were not thought to play a role in this function until now [8].

### Functional Advantage of Longer Flagellomeres in Adults

A visual criterion to distinguish the sexes in mantises lies in the longer antennae in males, as in *Tenodera aridifolia, Tenodera angustipennis* and *Pseudomantis albofimbriata* [5]. Slifer reported that it might be due to the fact that males possess more flagellomeres than females in *Tenodera angustipennis* [28]. However, there was no measurement of the length of individual flagellomeres in any of the studies reporting a difference in the length of antennae. In the present study, we examined differences in length between the sexes for each corresponding flagellomere in adults. These differences were not uniform along the antennae: the greatest differences occur in the intermediate parts (β to ε), where a large number of grooved peg sensilla are located [8].

Natural selection shapes structures, giving individuals the best chances of survival and reproduction. In general, it refines structures playing a role in important functions such as feeding, reproduction and survival, and diminishes useless structures [29]. Many factors affect sexual dimorphism which relate to the many aspects of reproduction and lifestyle of the species [30], [31]. For example, a bigger size in males confers upon them a mating advantage when they compete for females because they possess a physical advantage during competitions and have easier access to females [32], [33]. An expansion of the antennal surface can provide a good field for the development of a larger number of sensilla. This enables an increase in the number of particular types of sensilla, such as those receiving information about food or sex pheromones. The increased complexity of the shape and the elongation of antennae are both strategies to expand the antennal surface on which sensilla locate. For example, male moths (*Bombyx* and *Manduca*) have well-developed antennae compared to females [1]. Their antennae are equipped with a large number of sex-pheromone-receptive sensilla to seek females in adulthood. In hemimetabolous species, an expansion of the antennal surface might be more related to an elongation of antennae during postembryonic development. The present study may confirm this hypothesis in the sense that the majority of flagellomeres with the most expanded surfaces in males are related to those with a large number of grooved peg sensilla, a type of sensillum assumed to be involved in sex-pheromone reception [5], [34]. In contrast, there are no intersexual differences of antennal size in insects that do not use sex pheromones, such as some butterflies (*Papilionoidea*) [35], [36].

On the other hand, certain species of insects using sex pheromones for mating behavior have antennae of similar length and shape in both sexes, such as the cockroach *Periplaneta americana.* In cockroaches (*Periplaneta americana*), males detect sex pheromones released by females, but the antennae of both sexes are almost identical on the surface [17]. Their antennal dimorphism lies in more abundant sensilla responding to sex-pheromones in males, which appear during the final molting [37]. Although the cockroaches use sex pheromones during mating behavior, males have to search for females only over a short distance because of their aggregated lifestyle. Thus, an expansion of the surface of antennae might provide a critical advantage in species using olfaction to detect a distant sexual partner; a point that may be worthy of future research.

## Supporting Information

Appendix S1 **Detailed description of the antennal development between each instar.**
(DOCX)

Figure S1 **Lengths of flagellomeres in nymphs from the 1^st^ to the 5^th^ instars.** Graphs of comparisons of curves of lengths between the different instars (A: instars #1 and #2; B: instars #2 and #3; C: instars #3 and #4; D: instars #4 and #5) by normalizing the start of counting of the flagellomeres from the proximal (left) and distal (right) parts of the antennae. The flagellomeres are measured every 5 segments. The expansions of the different parts are shown in each graph.
(TIF)

Figure S2 **Lengths of flagellomeres in nymphs from the 5^th^ to 7^th^ instars (A and B: instars #5 and #6; C and D: instars #6 and #7) for males (A and C) and females (B and D).**
(TIF)

Figure S3 **Models to analyse the antennal expansion during the postembryonic development from the curves of length. A:** This figure represents the features used on the curves of lengths to separate the different part of the flagellum. Different models may be applicable from the movement of these features: starting counting the flagellomeres from the proximal part of the antennae, a right shift of the antipeak represents an increase in parts α and/or β (model A) **(B)**. In contrast, by counting from the distal part, a left shift represents an expansion of parts γ and/or δ (model B) **(C)**. **B:** In model A, new segments can be added to the part α (model 1) or to the part β (model 2). **C:** In model B, a left shift of the antipeak without movement of the black triangle represents an expansion of the part γ (model 3), while the left movement of the black triangle represents an expansion of the part δ (model 4).
(TIF)
