# Supplementary material for: Antennal Development in the Praying Mantis (Tenodera aridifolia) Highlights Multitudinous Processes in Hemimetabolous Insect Species
Source: PLoS One. 2014 Jun 4;9(6):e98324. doi: 10.1371/journal.pone.0098324 (PMC4045715; doi:10.1371/journal.pone.0098324)
Supplement: Appendix S1 — Detailed description of the antennal development between each instar. (DOCX) [file pone.0098324.s001.docx]

In the supplemental figures 1-2, the black triangle was positioned by noting the number of the flagellomeres transiting between the parts γ and δ, in other words the number of the flagellomere on which olfactory sensilla appeared. From this number, we easily calculated the number of flagellomeres composing part δ in relation to the total number of flagellomeres in each antenna.

Instars #1 to #2: After hatching, the nymphs had about 48.3 ± 1.6 flagellomeres (n = 11). During the first molting, the number of flagellomeres increased by about 6 (instar #2: 54 ± 0.6, t_12.805_ = -3.246, p = 0.006) (table 2) and was correlated to a simple right shift of the valley following the model A (figure S1A). Moreover, looking at the slope of the curve, the flagellomeres in the part β increased by 6 but those in the part α did not change in number (model 2). Thus, we concluded that 6 new flagellomeres were provided from the meriston to the part α and simultaneously 6 segments of the part α in the 1^st^ instar morphologically changed and adopted the features of the part β during the first molting.

Instars #2 to #3: Between these two instars, a total of 8 flagellomeres were added (instar #3: 62 ± 0.8, t_26_ = -8.074, p < 0.001). From the movements of the valley fitting the models 1 and 3, we considered that the parts α and γ each increased by about 4 flagellomeres (figure S1B). Only in this case, we observed a reduction of the length of the most proximal flagellomeres (e.g. model 1) between two successive instars. Concretely, the fact that 4 flagellomeres were added in the part γ suggested that two distal flagellomeres of the part β made a binary fission to 4 flagellomeres, which changed to adopt the features of those of the part γ. Simultaneously, 2 flagellomeres had to mature from the part α to those of the part β to keep the number of flagellomeres of the part β constant. Finally, 6 new flagellomeres had to be newly formed from the division of the meriston to compensate for the 2 segments that matured and by considering an expansion of the part α of about 4.

Instars #3 to #4: Between the 3^rd^ and 4^th^ instars, we observed an expansion of about 12 flagellomeres (instar #4: 73.8 ± 1.2, t_26_ = -8.206, p < 0.001). Among them, the part γ expanded by about 10 flagellomeres coming from binary divisions of 5 distal flagellomeres of the part β (figure S1C). Therefore, 7 segments from the part α at the 3^rd^ instar matured to give 7 new segments in the part β at the 4^th^ instar: 5 flagellomeres in the part β underwent a binary division and we estimated an expansion of the part β of about 2 flagellomeres due to the move of a clear valley to the right. Simultaneously, the meriston provided 7 new flagellomeres and a new meriston between these two instars. From there, the number of flagellomeres generated by the meriston increased at each molt until the 6^th^ instar.

Instars #4 to #5: Between the 4^th^ and 5^th^ instars, nymphs’ antennae were expanded by about 16 flagellomeres (instar #5: 90.2 ± 1.3, t_27_ = -8.943, p < 0.001). Although the number of flagellomeres possessing olfactory sensilla remained constant from the 1^st^ to the 4^th^ instars (instar #1: 23.2 ± 1.8, instar #4: 22.7 ± 1.3, t_17.569_ = -0.219, p = 0.829), an expansion of the part δ (with the appearance of olfactory sensilla on flagellomeres that were deprived of them before) started from the 5^th^ instar (part δ: 33.2 ± 1, t_24_ = -6.729, p < 0.001) as represented by a left shift of the black triangle in model 4 (figure S1D). Concretely, 10 distal flagellomeres of the part γ developed olfactory sensilla and matured to those of the part δ. As the valley left-shifted in a similar manner to the black triangle, we assumed that the number of flagellomeres of the part γ did not change after molting. This resulted in 5 distal flagellomeres from the part β undergoing a binary division. Taking into account the model 2, the part β was expanded by about 6 flagellomeres. Consequently, this indicated that 11 flagellomeres matured from the part α, and the constant number of flagellomeres in the part α also indicated that the same number of flagellomeres was newly formed from the meriston.

Instars #5 to #6: sexual dimorphism in mantis was visible from the 6^th^ instar, and we found that the number of flagellomeres was higher in males than in females (males: 111.2 ± 1.7, females: 103.5 ± 1.1, t_9_ = 3.928, p = 0.003) (table 2). This means that males developed a larger number of flagellomeres (≈ 21) than females (≈ 13). In addition, the parts δ for each sex expanded in a different manner (t_9_ = -3.460, p = 0.007), about 13 and 9 flagellomeres morphologically changed by developing olfactory sensilla in males (part δ: 46.2 ± 0.9, t_19_ = 7.175, p < 0.001) and females (part δ: 41.7 ± 0.9, t_20_ = 7.175, p < 0.001), respectively. However, looking at the curves (figures S2A-B right) and from the viewpoint of the models 3 and 4, the valley left-shifted only about 10 and 5 segments, respectively, in males and females, which is less than the expansion of the parts δ. This means that the parts γ reduced in both sexes. In order to know how the parts γ were reduced, we took into account the expansions appearing proximally (figures S2A-B left). We found that the parts β increased by about 10 and 8 flagellomeres in males and females, respectively, involving a respective reduction of 2 and 4 flagellomeres in the parts γ. In addition, there were more newly generated flagellomeres from the meriston in males (15) than in females (10) at the 6^th^ instar.

Instars #6 to #7: Once again, males developed more flagellomeres (increase of 26 compared to instar #6, t_13_ = -5.055, p < 0.001) than females (increase of 22, t_13_ = -4.717, p = 0.002) during this molting. In both sexes, our measurements showed an expansion of the parts δ (11, t_5.423_ = -2.347, p = 0.062 in males; and 16, t_13_ = -3.155 in females, p = 0.008) and of the parts γ in a higher manner (figures S2C-D). In return, the number of flagellomeres in the parts β was reduced in both sexes (15 segments in males and 10 in females). Finally, we counted that 5 (in males) and 6 (in females) flagellomeres were newly formed at the parts α from the division of the meriston.

Instars #7 to Adults: During the last molting, we found that there were no increase in the number of flagellomeres in males (instar #7: 137.2 ± 3.5, adults: 130.7 ± 2.9, t_15_ = 1.345, p = 0.199) and females (instar #7: 125.2 ± 4.5, adults: 121.4 ± 0.9, t_8.636_ = 0.831, p = 0.428). The mean number of flagellomeres tended to decrease in adults for both sexes, probably due to the amputation of the tip end of antennae during the last molting. This phenomenon has been already reported in cockroaches [[1](#_ENREF_1)]. Before this last transition, the sexual dimorphism concerned only the number of flagellomeres and their width (see results). Nevertheless, during the last molting, males also developed a large number of grooved peg sensilla and longer flagellomeres compared to females.

**References**

1. Schafer R, Sanchez TV (1973) Antennal sensory system of the cockroach, Periplaneta americana: postembryonic development and morphology of the sense organs. Journal of Comparative Neurology 149: 335-354.
