# Supplementary figures and images for: Antennal Development in the Praying Mantis (Tenodera aridifolia) Highlights Multitudinous Processes in Hemimetabolous Insect Species
Source: PLoS One. 2014 Jun 4;9(6):e98324. doi: 10.1371/journal.pone.0098324 (PMC4045715; doi:10.1371/journal.pone.0098324)

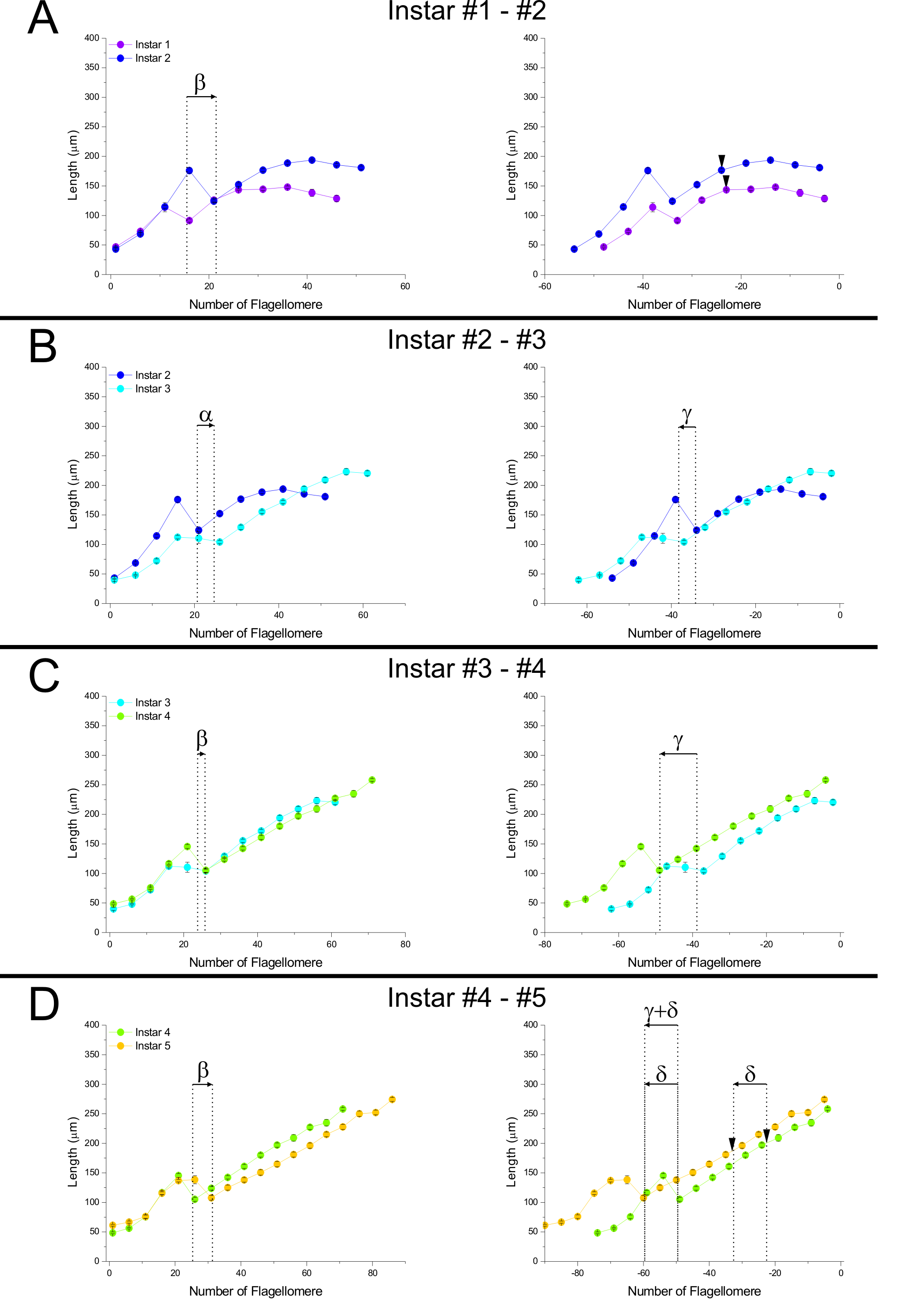

Supplement: Figure S1 — Lengths of flagellomeres in nymphs from the 1st to the 5th instars. Graphs of comparisons of curves of lengths between the different instars (A: instars #1 and #2; B: instars #2 and #3; C: instars #3 and #4; D: instars #4 and #5) by normalizing the start of counting of the flagellomeres from the proximal (left) and distal (right) parts of the antennae. The flagellomeres are measured every 5 segments. The expansions of the different parts are shown in each graph. (TIF) [file pone.0098324.s002.tif]

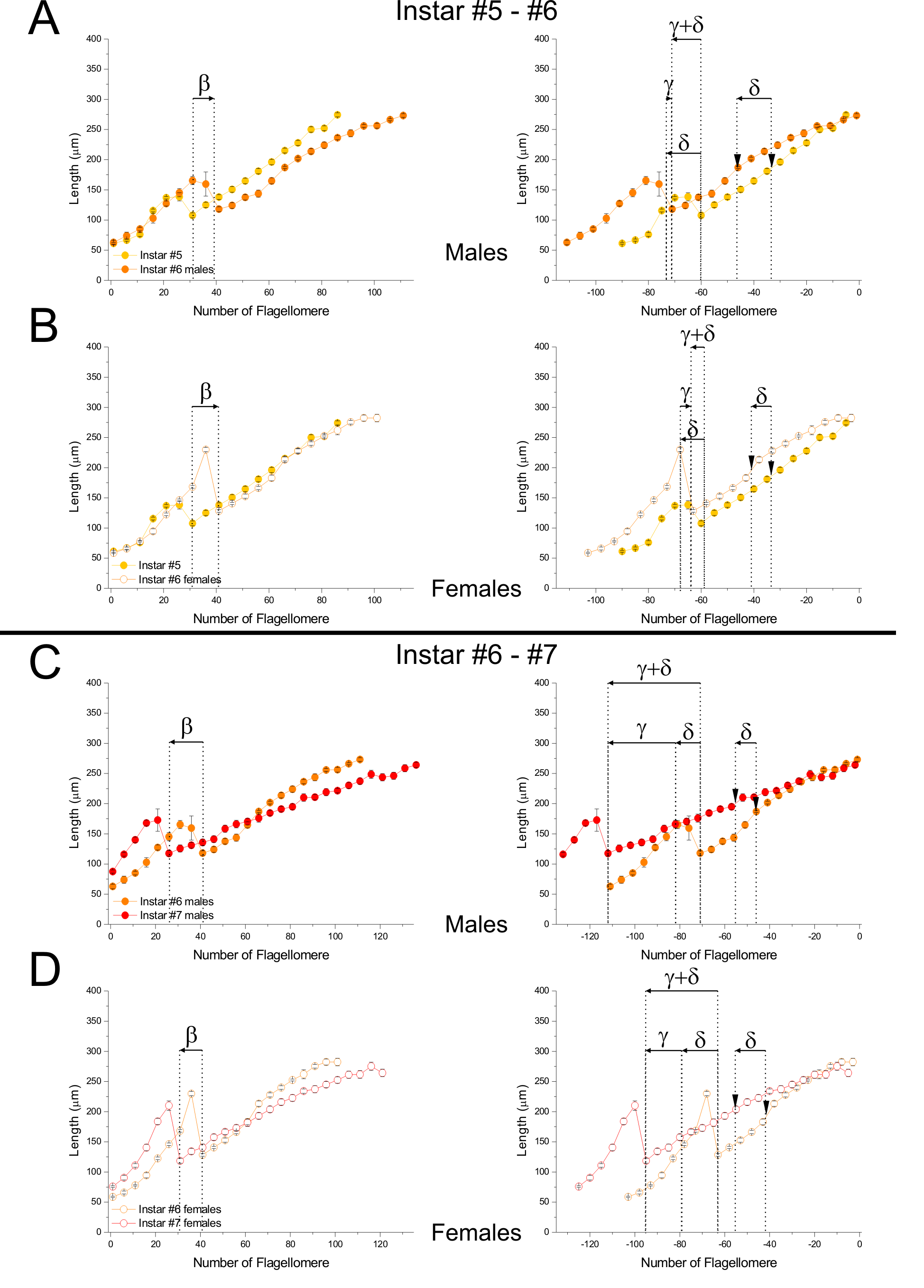

Supplement: Figure S2 — Lengths of flagellomeres in nymphs from the 5th to 7th instars (A and B: instars #5 and #6; C and D: instars #6 and #7) for males (A and C) and females (B and D). (TIF) [file pone.0098324.s003.tif]

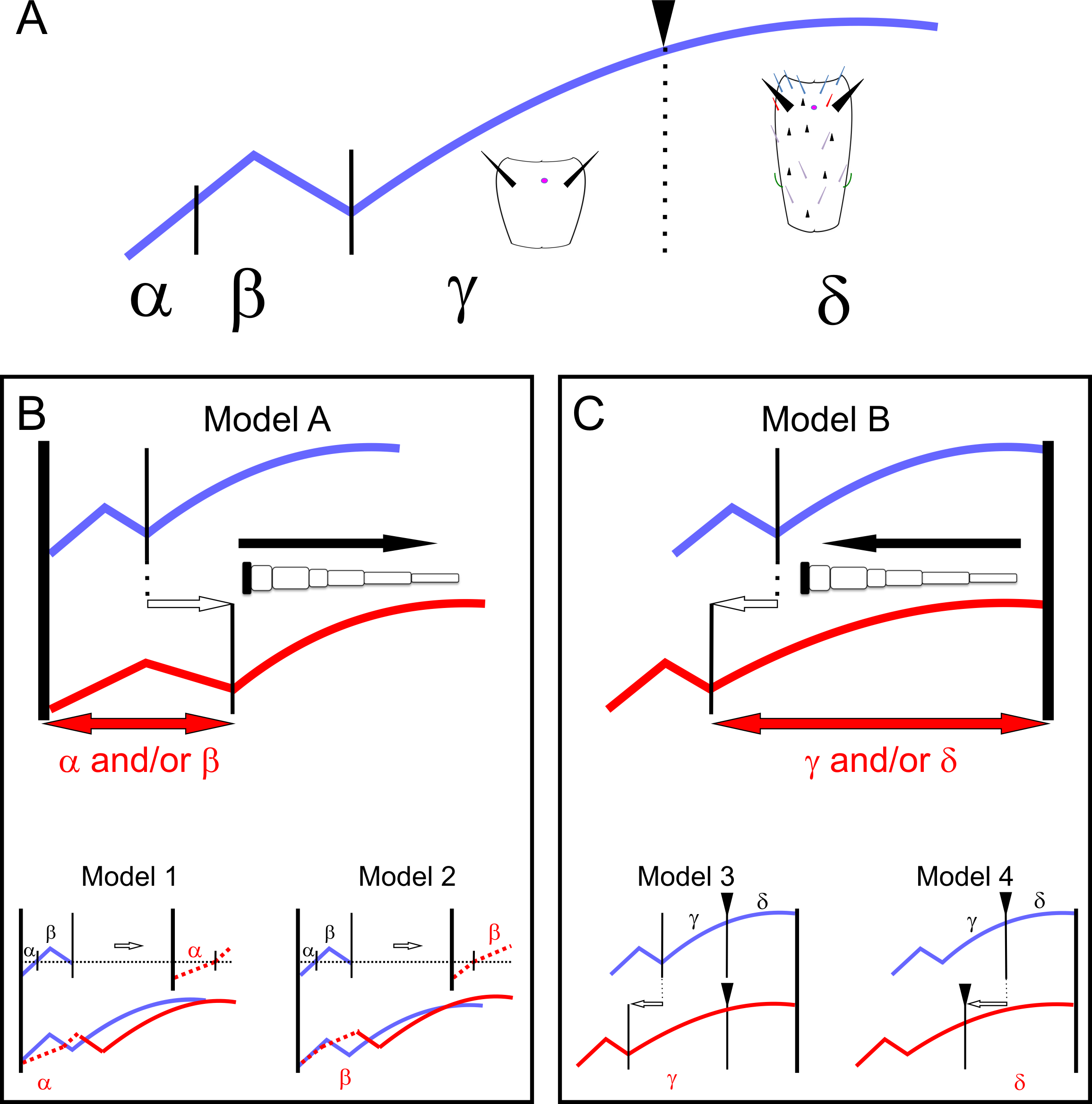

Supplement: Figure S3 — Models to analyse the antennal expansion during the postembryonic development from the curves of length. A: This figure represents the features used on the curves of lengths to separate the different part of the flagellum. Different models may be applicable from the movement of these features: starting counting the flagellomeres from the proximal part of the antennae, a right shift of the antipeak represents an increase in parts α and/or β (model A) (B). In contrast, by counting from the distal part, a left shift represents an expansion of parts γ and/or δ (model B) (C). B: In model A, new segments can be added to the part α (model 1) or to the part β (model 2). C: In model B, a left shift of the antipeak without movement of the black triangle represents an expansion of the part γ (model 3), while the left movement of the black triangle represents an expansion of the part δ (model 4). (TIF) [file pone.0098324.s004.tif]
